# Supplementary material for: Improving the specificity of nucleic acid detection with endonuclease-actuated degradation
Source: Commun Biol. 2022 Mar 31;5:290. doi: 10.1038/s42003-022-03242-x (PMC8971390; doi:10.1038/s42003-022-03242-x)
Supplement: Supplementary file 2 — Supplementary Information [file 42003_2022_3242_MOESM2_ESM.pdf]

Improving the specificity of nucleic acid detection with endonuclease-actuated degradation

Roger S. Zou[1,2], Momcilo Gavrilov[2,3], Yang Liu[2], Dominique Rasoloson[4,5], Madison Conte[6], Justin P. Hardick[6], Leo Shen[1,2], Siqi Chen[1,4], Andrew Pekosz[7], Geraldine Seydoux[4,5], Yukari C. Manabe[6], Taekjip Ha[1,2,3,5,*]

1 Department of Biomedical Engineering, Johns Hopkins University School of Medicine, Baltimore, Maryland, USA
2 Department of Biophysics and Biophysical Chemistry, Johns Hopkins University School of Medicine, Baltimore, Maryland, USA
3 Department of Biophysics, Johns Hopkins University, Baltimore, Maryland, USA
4 Department of Molecular Biology and Genetics, Johns Hopkins University School of Medicine, Baltimore, Maryland, USA
5 Howard Hughes Medical Institute, Baltimore, Maryland, USA
6 Division of Infectious Diseases, Department of Medicine, Johns Hopkins University School of Medicine, Baltimore, Maryland, USA
7 Department of Molecular Microbiology and Immunology, Johns Hopkins University Bloomberg School of Public Health, Baltimore, Maryland, USA

* Correspondence: tjha@jhu.edu

Supplementary Figures 1-4
Supplementary Tables 1-2

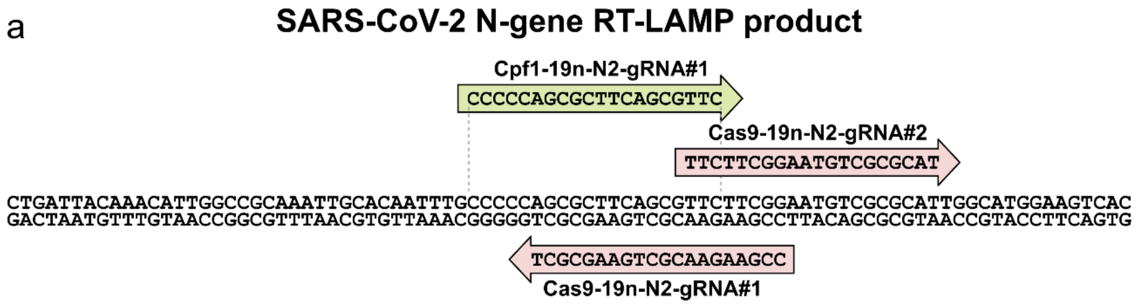

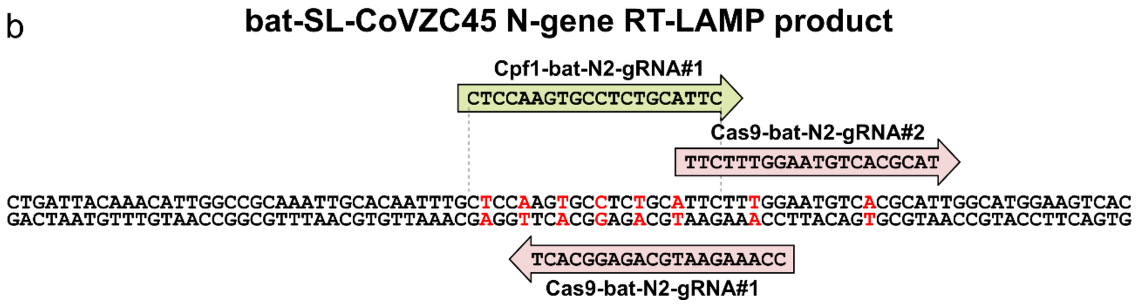

Supplementary Figure 1: Template and guide RNA sequences

Sections of (a) SARS-CoV-2 and (b) bat-SL-CoVZC45 N-gene sequences, amplified by LAMP primers, that contain endonuclease targeting sites. The two Cas9 target sequences (Cas9-19n-N2-gRNA#1, Cas9-19n-N2-gRNA#2) and one AsCpf1 target sequence (Cpf1-19n-N2-gRNA#1) are shown.

a

$$\left(1-\frac{A}{B}\right)\times\frac{B}{C}$$

(+) $$\left(1-\frac{15841}{35802}\right)\times\frac{35802}{2961}=6.74$$

(−) $$\left(1-\frac{35183}{38627}\right)\times\frac{38627}{2961}=1.16$$

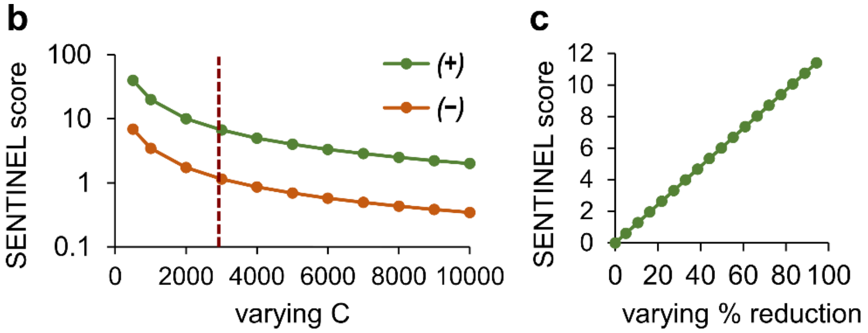

Supplementary Figure 2: Analysis and interpretation of SENTINEL scores

a) Formula for SENTINEL score (top row). Calculation of SENTINEL scores for samples that both amplify with LAMP, and have the expected target sequence for Cas9/gRNA cleavage *(+),* or is not cleaved with Cas9/gRNA *(−)*. Note that the difference in scores (6.74 vs 1.16) is mainly driven by the difference in A between the + and − reactions, given that B and C are similar in their respective + and − reactions.

b) Perturbation analysis by varying the variable C, keeping other variables the same as panel a. The vertical red dotted line corresponds to the values from panel a.

c) Perturbation analysis by varying the variable A relative to B (A/B), keeping other variables the same as panel a.

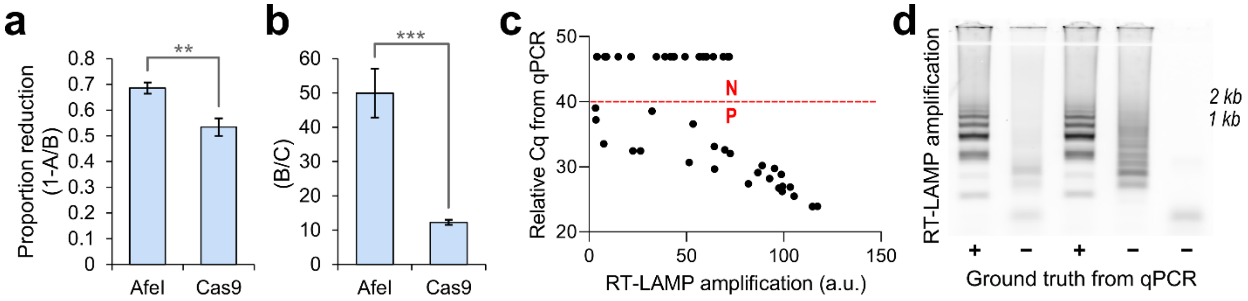

Supplementary Figure 3: Comparison between endonucleases, and evaluation of patient nasopharyngeal swabs

a-b) Values of individual components of the SENTINEL score, (a) 1-A/B and (b) B/C, using AfeI versus Cas9 as the endonuclease. Error bars represent ±1 standard deviation from mean for 3 replicates. ** indicates $p<0.01$, *** indicates $p<0.001$ from Student's t-test.

c) Graph of RT-LAMP product quantification (measured by PicoGreen dsDNA fluorescence, arbitrary units) versus Cq from qPCR for the 50 blinded patient samples (25 with detectable virus from qPCR – region below dotted line labeled with 'P', i.e., Cq below 40. 25 negative controls – region above dotted line labeled with 'N', i.e., Cq above 40). qPCR Cqs were initially measured from 300 µL of patient sample, and adjusted here to be consistent with the 2.5 µL of patient samples used for SENTINEL by adding $\log_2(300/2.5) = 6.9$.

d) RT-LAMP product directly from patient nasopharyngeal (NP) swabs, on agarose gel electrophoresis. Samples expected to have virus in lanes 1 and 3 from left (labeled with '+'). Samples not expected to have virus in other lanes (labeled with '–'). Note that non-specific RT-LAMP amplification occurs for some virus-negative patient NP swabs – lanes 2 and 4 from left. 1 kb and 2 kb size markers are labeled on the right.

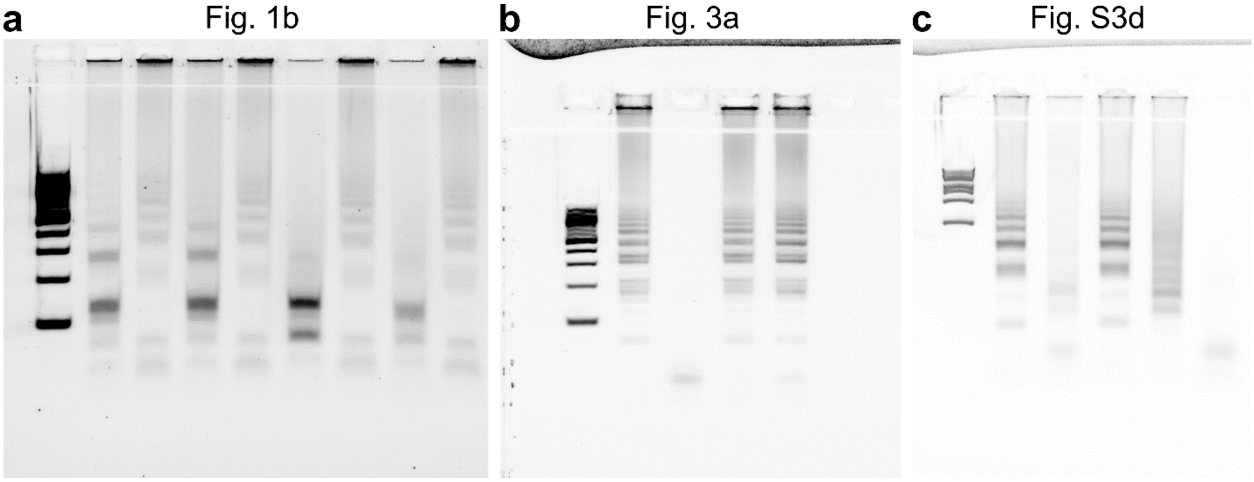

Supplementary Figure 4: Raw gel images

Raw gel images for (a) Fig. 1b, (b) Fig. 3a, and (c) Fig. S3d.

Supplementary Table 1: Oligonucleotide sequences

- Asterisk (*) corresponds to phosphorothiolate modifications

Name	Sequence
IVT_19n_N2_FWD	AATTCTAATACGACTCACTATAGGGCCAAATTGGCTACTACCGAAGAGCTAC
IVT_bat_N2_FWD	AATTCTAATACGACTCACTATAGGGCCAAATTGGCTACTACCGTAGAGCTAC
IVT_19n_N2_REV	CACAGTTTGCTGTTTCTTCTGTCTCTG
IVT_bat_N2_REV	CACAGTTTGTTGTTTCTTCTGTCTCTGC
RTL_19n/bat_N2_FIP-p	T*G*C*G*G*CCAATGTTTGTAATCAGCCAAGGAAATTTTGGGGAC
RTL_19n_N2_BIP-p	C*G*C*A*T*TGGCATGGAAGTCACTTTGATGGCACCTGTGTAG
RTL_bat_N2_BIP-p	C*G*C*A*T*TGGCATGGAAGTCACtttaatggctccatgataa
RTL_19n_N2_F3-p	A*A*C*A*C*AAGCTTTCGGCAG
RTL_bat_N2_F3-p	c*a*c*t*c*aagcatttgggag
RTL_19n_N2_B3-p	G*A*A*A*T*TTGGATCTTTGTCATCC
RTL_bat_N2_B3-p	g*a*a*t*t*gtggatctttgtcatcc
RTL_19n_N2_LF-p	T*T*C*C*T*TGTCTGATTAGTTC
RTL_bat_N2_LF-p	t*t*c*c*t*tgtctgattaattc
RTL_19n_N2_LB-p	A*C*C*T*T*CGGGAACGTGGTT
RTL_bat_N2_LB-p	a*c*c*t*t*cgggaacatggct
RTL_19n_N2_F3-p	A*A*C*A*C*AAGCTTTCGGCAG
RTL_bat_N2_F3-p	c*a*c*t*c*aagcatttgggag

Supplementary Table 2: guide RNA protospacer sequences

**Name**	**Protospacer sequence**
Cas9-19n-N2-gRNA#1	CCGAAGAACGCTGAAGCGCT
Cas9-bat-N2-gRNA#1	CCAAAGAATGCAGAGGCACT
Cas9-19n-N2-gRNA#2	TTCTTCGGAATGTCGCGCAT
Cas9-bat-N2-gRNA#2	TTCTTTGGAATGTCACGCAT
Cpf1-19n-N2-gRNA#1	CCCCCAGCGCTTCAGCGTTC
Cpf1-bat-N2-gRNA#1	ctccaagtgcctctgcattc
Cpf1-negctrl-gRNA#1	CGTTAATCGCGTATAATACGG

* Cas9 negative control is commercially purchased as Alt-R® CRISPR-Cas9 Negative Control crRNA #1 (Integrated DNA Technologies 1072544)
