# Supplementary material for: Improving the specificity of nucleic acid detection with endonuclease-actuated degradation
Source: Commun Biol. 2022 Mar 31;5:290. doi: 10.1038/s42003-022-03242-x (PMC8971390; doi:10.1038/s42003-022-03242-x)
Supplement: Supplementary file 3 — Description of Additional Supplementary Files [file 42003_2022_3242_MOESM3_ESM.pdf]

Description of Additional Supplementary Files

File name: Supplementary Data 1
Description: Raw data for figures.
